# Mid-term Efficacy of Local Repair Using Modified Altemeier Technique for Stomal Prolapse: A Case Series

**DOI:** 10.7759/cureus.28193

**Published:** 2022-08-20

**Authors:** Shingo Tsujinaka, Nao Kakizawa, Yuuri Hatsuzawa, Ryo Maemoto, Natsumi Matsuzawa, Sawako Tamaki, Yuji Takayama, Yasuyuki Miyakura, Toshiki Rikiyama

**Affiliations:** 1 Surgery, Saitama Medical Center, Jichi Medical University, Saitama, JPN; 2 Surgery, Saitama Medical Center, Jichi Medical Univeristy, Saitama, JPN

**Keywords:** mid-term results, recurrence, stomal prolapse, local repair, altemeier technique

## Abstract

Introduction: Stomal prolapse (SP) is characterized by full-thickness protrusion of the bowel through the stoma site. The surgical procedures for SP include local repair, abdominal wall fixation, and stoma relocation. However, previous reports were mostly case reports or case series with a small number of patients and lacked long-term results. A modified Altemeier technique (MAT) has been used for the local repair of SP in our institution, and this study aimed to evaluate its mid-term efficacy.

Methods: We reviewed patients who underwent MAT for SP between August 2013 and December 2020. The variables included patient characteristics, type of stoma, indications of stoma creation, the time interval from stoma creation to prolapse, site of prolapse, reasons for SP surgery, perioperative variables, complications during SP surgery, and length of follow-up. Recurrence of SP was defined as the need for change in stoma care or re-protrusion of the stoma by more than 5 cm in length.

Results: Ten patients were included in this study. The median age at the time of SP surgery was 71.5 years. The indications of stoma creation included unresectable or recurrent intra-abdominal malignancies in four patients, diverting ileostomy with rectal cancer surgery in two, transverse colon cancer in one, gastric and rectal cancer in one, rectovaginal fistula in one, and non-occlusive mesenteric ischemia in one. The median interval from stoma creation to prolapse was 2.5 months. Six patients underwent elective SP surgery, and four patients underwent emergency surgery for incarcerated prolapse. The median operative time was 75.5 min. Postoperative complications that included transient mucosal ischemia and subcutaneous abscess occurred in one patient. There were four recurrences (40%), and the median time interval from surgery to recurrence was 4.5 months. Two patients underwent repeated MAT, one of whom underwent stomal reversal with laparotomy for re-recurrence. The median follow-up duration was 19 months.

Conclusion: MAT for SP is associated with a high recurrence rate in mid-term follow-up.

## Introduction

Intestinal stoma is considered one of the most common surgical procedures worldwide. Stoma creation is indicated in patients with a variety of benign or malignant gastrointestinal diseases, and it may be performed in either emergency or elective settings [1]. Stomal prolapse (SP) is characterized by full-thickness protrusion of the bowel through the stoma site, and its incidence is reported to be 7%-26% [2]. SP typically occurs as a late complication after stoma surgery (after six months), and the distal limb is mostly affected [2]. The symptoms of SP include skin irritation, mucosal bleeding, bowel ischemia, incarceration, and difficulty in managing stoma appliances [3-4].

Surgery is indicated when the patient is resistant to nonsurgical, conservative treatment such as manual reduction, dietary therapy, bowel management, and revision of the stoma appliance. The surgical procedures include local repair, abdominal wall fixation, and stoma relocation. However, previous reports were mostly case reports or case series with a relatively small number of patients [5-12]; therefore, mid- or long-term results are lacking. In 2020, one of the largest case series (23 cases) was published, and the recurrence rate was > 40% after either local repair or laparotomy [13]. In 2021, Koide et al. reported excellent results after local repair using a stapler device, where only one out of 24 patients experienced recurrence [14]. However, this procedure may require multiple firings of the staples, resulting in increased surgical costs.

The Altemeier technique is a well-known transperineal procedure for full-thickness rectal prolapse [15]. A modification of this technique, the modified Altemeier technique (MAT), has been used for local repair of SP [11-12]. This procedure is technically simple, consists of prolapsed bowel resection with anastomosis using sutures alone, and is applicable by surgeons with any level of expertise. This study aimed to evaluate the mid-term efficacies of MAT at our institution.

## Materials and methods

We reviewed patients who underwent MAT for SP between August 2013 and December 2020 in our institute. All data were collected from electronic medical charts. The variables included patient characteristics [age, sex, and American Society of Anesthesiologist-Physical Status (ASA-PS)], type of stoma, indications of stoma creation, time interval from stoma creation to prolapse, site of prolapse, reasons for SP surgery, perioperative variables during SP surgery (timing, operative time, and estimated blood loss), postoperative complications according to the Clavien-Dindo classification [16], and the length of follow-up. Recurrence of SP was defined as the need for change in stoma care or re-protrusion of the stoma by more than 5 cm in length [17].

**Figure 1:**
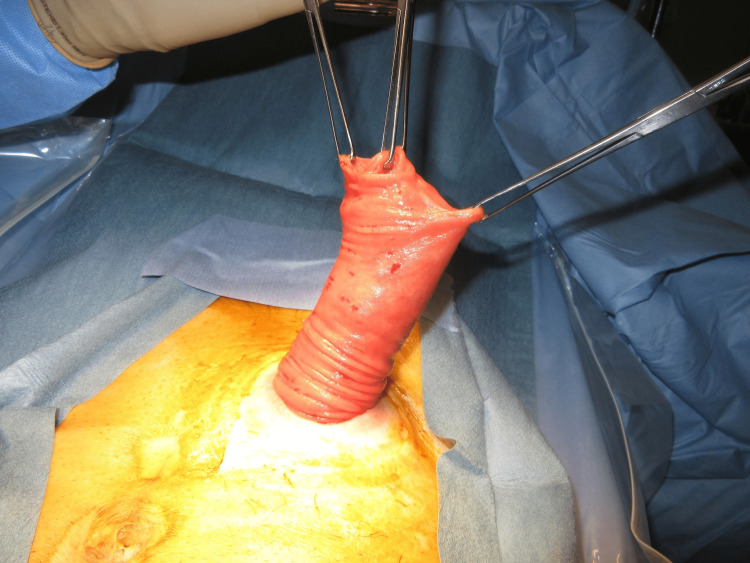
Maximal inversion of the prolapsed limb.

**Figure 2:**
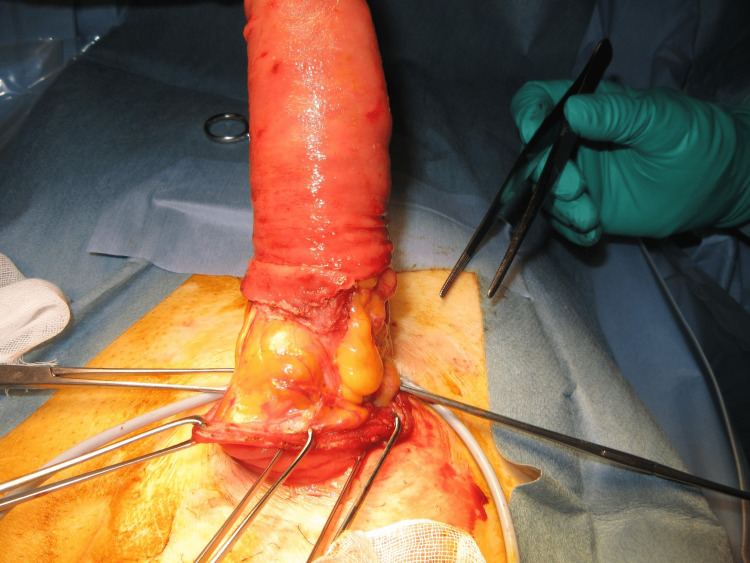
A circumferential, full-thickness incision of the prolapsed limb.

**Figure 3:**
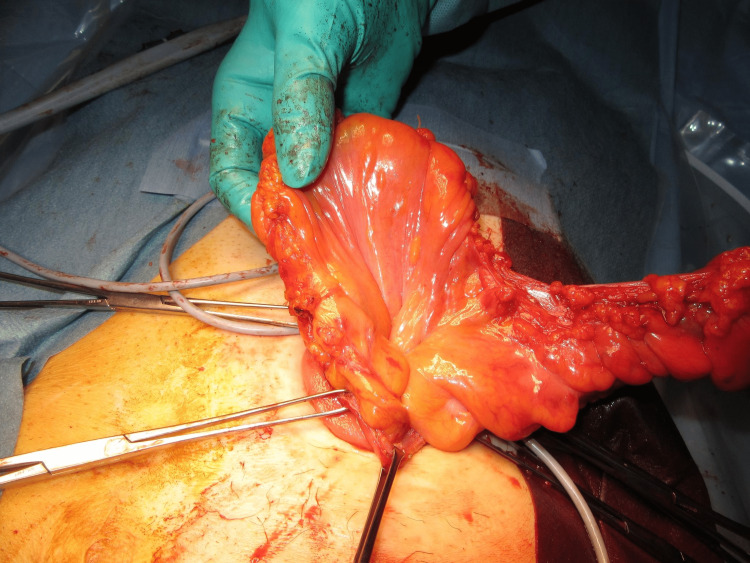
Ligation and division of the mesentery.

**Figure 4:**
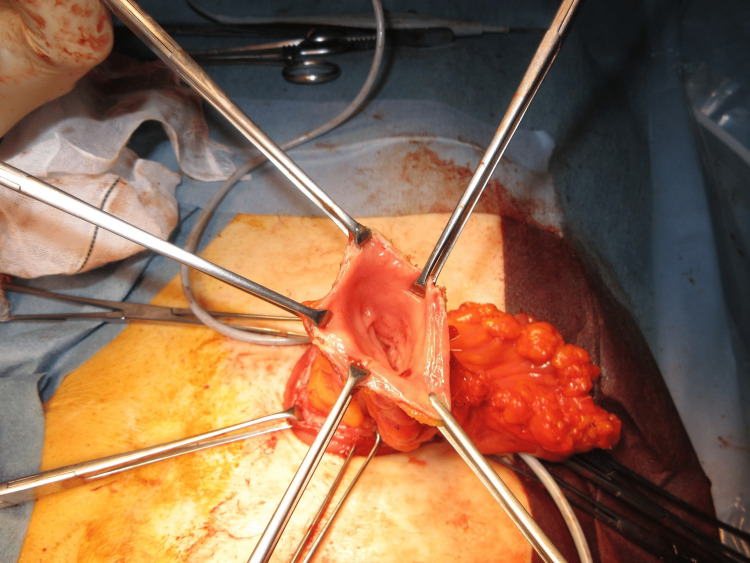
Complete division of the prolapse limb.

**Figure 5:**
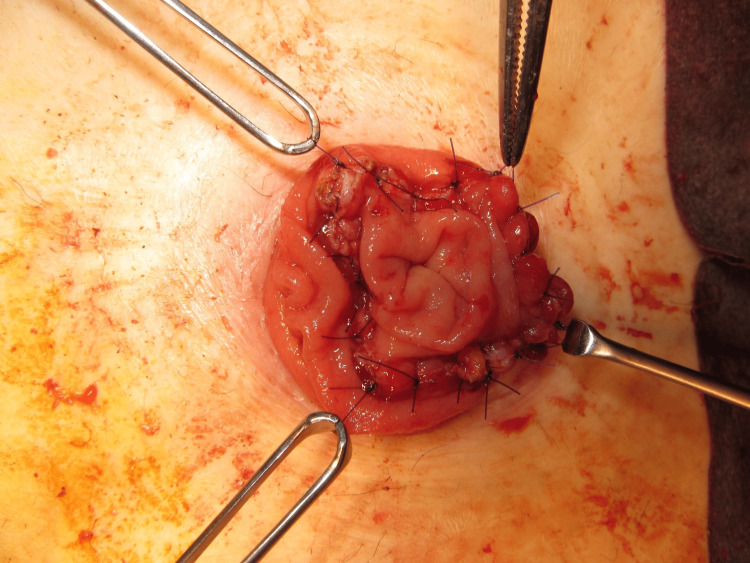
Handsewn anastomosis between the proximal and distal bowel.

**Figure 6:**
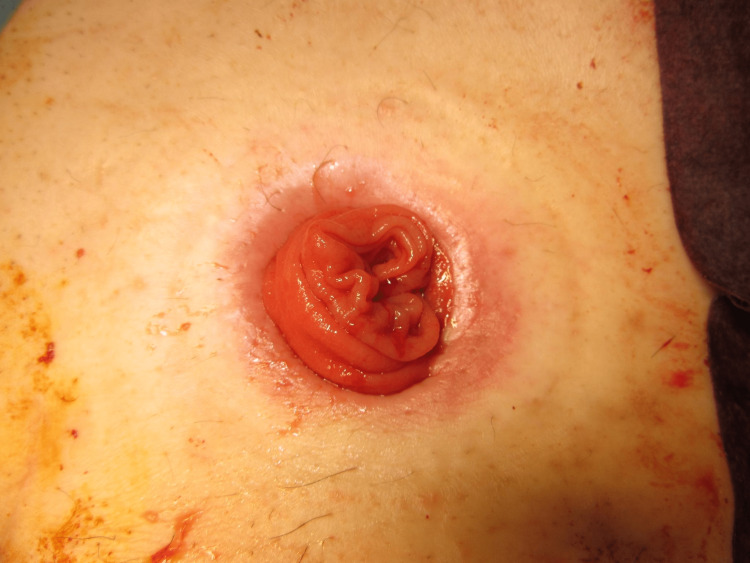
Reduction of the revised stoma.

The MAT was performed according to a previously described technique [12]. The prolapsed limb was inverted and maximally pulled out with the patient under general anesthesia and in the supine position (Figure 1). A circumferential, full-thickness incision was made 2 cm above the mucocutaneous junction (Figure 2). The mesentery was then ligated and divided to avoid injuring the blood supply to the distal edge of the limb at the mucocutaneous junction (Figure 3). After the complete division of the prolapsed bowel (Figure 4), hand-sewn anastomosis was performed between the proximal and distal edges of the bowel using a 4-0 absorbable suture. Typically, 16-20 full-thickness stitches are applied (Figure 5). Finally, the anastomosed bowel was everted and reduced (Figure 6). In cases of simultaneous prolapse of the distal and proximal limbs, both sides of the SP were repaired using the same procedure.

 

 

 

 

 

This study was approved by the Bioethics Committee for Clinical Research of Saitama Medical Center, Jichi Medical University (#S20-229). Because this was a retrospective study, written consent was not obtained from the patients in advance. Instead, information about the research was disclosed through our hospital website in accordance with the “opt-out method.”

## Results

Ten patients were identified, and two underwent repeated MAT. Table 1 summarizes the patients’ characteristics and information regarding their stomas. There were six men and four women, and the median age at SP surgery was 71.5 years (range 58-78 years). The indications of stoma creation included unresectable or recurrent intra-abdominal malignancies in four patients, diverting ileostomy with rectal cancer surgery in two, transverse colon cancer in one, gastric and rectal cancer in one, rectovaginal fistula in one, and non-occlusive mesenteric ischemia in one. The types of stoma included loop colostomy in five patients, loop ileostomy in two, end colostomy in two, and end ileostomy in one. The median time interval from stoma creation to prolapse was 2.5 months (range 1-11 months). The prolapse site was the distal limb in five patients and both the proximal and distal limbs in two patients with loop stoma.

**Table 1:** Patient characteristics and information regarding their stomas. ASA-PS, American Society of Anesthesiologist-physical status; N/A, not applicable

Case	Age	Gender	ASA-PS	Indication of stoma creation	Type of stoma	Stoma location	Time interval to prolapse (months)	Site of prolapse
1	67	Male	2	Recurrent gastric cancer	Loop	Sigmoid colon	1	Distal
2	75	Male	3	Diverting ileostomy with rectal cancer	Loop	Ileum	2	Proximal and distal
3	78	Female	3	Rectovaginal fistula	Loop	Transverse colon	8	Distal
4	70	Female	2	Recurrent ovarian cancer	Loop	Ileum	2	Proximal and distal
5	70	Female	2	Recurrent ovarian cancer	Loop	Transverse colon	1	Distal
6	58	Female	2	Unresectable rectal cancer	Loop	Transverse colon	11	Distal
7	75	Male	2	Non-occlusive mesenteric ischemia	End	Sigmoid colon	10	N/A
8	72	Male	3	Transverse colon cancer	End	Ileum	2	N/A
9	71	Male	2	Gastric and rectal cancer	End	Sigmoid colon	3	N/A
10	75	Male	2	Diverting ileostomy with rectal cancer	Loop	Transverse colon	3	Distal

Table 2 shows the reasons, timing of the SP surgery, and treatment outcomes.　Six patients underwent elective SP surgery, and four patients underwent emergency surgery for incarcerated prolapse. The median operative time was 75.5 min (range 45-105 min) and the median estimated blood loss was 10 mL (range 10-70 mL). Postoperative complications occurred in one patient (Case 10). The patient had transient mucosal ischemia (Clavien-Dindo II) and subcutaneous abscess (Clavien-Dindo IIIa). There were four SP recurrences (40%), and the median time interval from surgery to recurrence was 4.5 months (range 2-13 months). Recurrence was observed in two palliative stomas with unresectable intra-abdominal malignancies: one diverting ileostomy with rectal cancer surgery and one end colostomy with non-occlusive mesenteric ischemia. Two patients underwent repeated MAT (Cases 8 and 10). One patient lived without recurrence until death (Case 10), but another patient experienced recurrent symptoms requiring ileocolic anastomosis as further intervention (Case 8). The median follow-up period was 19 months (range 7-49 months) and six patients died at the end (unresectable or recurrent cancer in five, uncertain cause in one).

**Table 2:** Reasons, timing of SP surgery, and treatment outcomes. SP, stomal prolapse; QOL, quality of life

Case	Reason for surgery	Postoperative complications	SP recurrence	Time to recurrence (months)	Follow-up length (months)
1	Incarceration	None	No	N/A	9
2	Incarceration	None	No	N/A	21
3	Reduced QOL	None	No	N/A	18
4	Reduced QOL	None	Yes	2	15
5	Incarceration	None	No	N/A	49
6	Reduced QOL	None	No	N/A	7
7	Incarceration	None	Yes	7	12
8	Reduced QOL	None	Yes	2	42
9	Reduced QOL	None	No	N/A	20
10	Reduced QOL	Mucosal ischemia and subcutaneous abscess	Yes	13	24

## Discussion

The factors responsible for SP have been suggested to be redundant intestine, mobile intestine, and the space between the abdominal wall and the intestine [14, 18]. For acute SP, the use of topical sugar may be effective when reducing the prolapsed intestine to avoid emergency surgery [19]. Redundant intestines can be shortened using Delorme’s technique [6, 9] or resected using MAT [11-12] or stapler devices [5, 7-8, 14, 17-18, 20]. The mobile intestine can be fixed using button-pexy [21], endoscopic-assisted procedures [22], or laparoscopic enteropexy [23]. The space between the abdominal wall and intestine can be narrowed by local treatment using a mesh strip technique [24] or Thiersch’s technique [10]. The advantage of local repair appears to be its simplicity and applicability as a first-line treatment, without the need for laparotomy for laparoscopy under general anesthesia.

This study showed a relatively high recurrence rate of prolapse (40%) after MAT with a median follow-up of 19 months. The recurrence rate was consistent with one of the largest previous reports (43.5%) consisting of various surgical approaches with a median follow-up of 24 months. In contrast, Papadopoulos et al. reported a case of MAT with no recurrence after 24 months, and Watanabe et al. reported three cases with no recurrence after 6-20 months [11-12]. The higher recurrence rate in this study might be due to the heterogeneity in patients’ backgrounds, indications of surgery, or details of the surgical technique; however, the small number of patients made it difficult to identify specific factors associated with recurrence [13].

For the local repair, the use of a stapler device has been reported to result in a low recurrence rate. Koide et al. demonstrated that there was only one recurrence among 24 patients who underwent local repair using linear staplers [14]. No recurrence has been previously reported using a similar technique [5, 7-8, 14]. Recently, another local repair method using a linear stapler was proposed, named “Phillips correction,” for prolapsed end ileostomy; in this method, a repeated procedure was successfully performed in a recurrent case [20]. Moreover, the median operative time was reportedly shorter in the stapler technique than in MAT (20-40 min [8, 14] vs. 75.5 min in this study). One of the potential disadvantages of stapler devices is the increased surgical cost associated with the use of multiple firing. In some countries, the cost of staples is not covered by health insurance [7-8, 12, 14, 17-18]. In general, local repair was associated with no or very low rates of postoperative complications in all studies [5, 7-14, 17, 20, 22, 24], suggesting that surgery can be performed in a minimally invasive manner. The choice of surgical procedure may be considered based on the relevant symptoms and pathophysiology of SP, such as redundant intestine, mobile intestine, or the space between the abdominal wall and the intestine.

In this study, two patients underwent repeated MAT for recurrent SP. However, one patient experienced re-recurrence and underwent subsequent laparotomy with stoma reversal. Mittal et al. reported a similar re-recurrence rate (50%) after the local repair, in which one patient underwent subsequent laparotomy and another lived with SP [13]. The redo stapler technique can be successfully repeated for recurrent cases without re-recurrence [14, 20]. These results suggest that redo MAT should not be recommended for recurrent SP; therefore, local repair using a stapler device or other surgical approaches should be considered.

In this study, the time interval from surgery to recurrence was 4.5 months (range 2-13 months). Mittal et al. reported that recurrence was diagnosed at a median time of 7.5 months (range 1-51 months) after the initial SP repair, and eight out of 10 recurrences occurred within one year. SP surgery with redundant bowel resection alone may not be sufficient, and the surgical indication might be questionable, especially in patients with early recurrence (one or two months after repair). As such, the indication and timing of surgery and the benefits and risks of surgical repair for recurrent SP should be discussed between patients and surgeons.

The limitations of this study are its retrospective nature, a small number of patients, and single-arm surgical procedure without a control group. Moreover, the type of stoma and reason for stoma creation were heterogeneous among the patients. Given the lack of a large dataset and the possibly rare surgical indication for SP, prospective, multi-institutional studies are needed to assess the efficacy of any type of surgical repair for SP.

## Conclusions

The MAT for SP is associated with a high recurrence rate in mid-term follow-up. The MAT may be an optional treatment in selected patients with SP, considering the minimal risk of postoperative complications and reduced medical costs.
